# The neuropsychotropic effects of *Crocus sativus* L. (saffron): an overview of systematic reviews and meta-analyses investigating its clinical efficacy in psychiatric and neurological disorders

**DOI:** 10.22038/AJP.2021.19300

**Published:** 2022

**Authors:** Ahmad Shamabadi, Alireza Hasanzadeh, Shahin Akhondzadeh

**Affiliations:** 1 *School of Medicine, Tehran University of Medical Sciences, Tehran, Iran*; 2 *Psychiatric Research Center, Roozbeh Psychiatric Hospital, Tehran University of Medical Sciences, Tehran, Iran*

**Keywords:** Crocus sativus, Herbal medicine, Neurology, Pharmacognosy, Systematic review

## Abstract

**Objective:**

Saffron is a spice derived from the *Crocus sativus *L. with antioxidant, anti-inflammatory, and neuroprotective effects. This study aims to systematically review the systematic reviews (SRs) investigating the clinical neuropsychotropic effects of saffron.

**Materials and Methods:**

The protocol of this SR was registered in PROSPERO (CRD42021268446). Scopus, ISI Web of Science, Embase, MEDLINE, PubMed, CINAHL, Cochrane Library, Google Scholar, and PROSPERO were searched up to June 6, 2021, to find SRs investigating the neuropsychotropic effects of saffron. The primary outcome was a report on whether or not saffron was effective in each study. AMSTAR was checked for the included reviews.

**Results:**

Twenty-three studies were reviewed with a mean AMSTAR score of 6.08 (ranging from 1 to 10). Thirteen SRs investigated the effects of saffron on depression. Six of the SRs studied its impact on sexual dysfunction. Each of the anxiety and cognitive disorders was discussed in three distinct reviews. Furthermore, possible effects of saffron on some other disorders, like premenstrual syndrome, postpartum depression, sleep disorders, and snacking behavior, have been reported.

**Conclusion:**

Saffron is beneficial, safe, and tolerable in treating the mentioned neurological and psychiatric disorders. Further high-quality, large-scale studies are recommended to rectify the shortcomings.

## Introduction

Saffron is a species derived from the dried red stigmas of *Crocus sativus *L., which is from the iris family (Iridaceae) (Srivastava et al., 2010 ▶). The main secondary metabolites of saffron are crocin, crocetin, picrocrocin, and safranal, which have antioxidant, anti-inflammatory, and neuroprotective effects (Rameshrad et al., 2018 ▶).

Traditional medicine considers saffron a spice with nerve sedation and anticonvulsant effects (Mzabri et al., 2019 ▶). In the last two decades, an increasing number of *in-vitro*, animal, and clinical studies have reported its effectiveness in treating neurological and psychiatric disorders and have proposed possible mechanisms for them (Zandi et al., 2021 ▶).

To date, several systematic reviews (SRs) of these reports have been published. However, to the best of our knowledge, no study has documented, categorized, and reviewed the evidence generated by these SRs and assessed their quality. This study aimed to systematically identify, select, and appraise, for the first time, the SRs investigating the neuropsychotropic effects of saffron and its effectiveness in the treatment of neurological and psychiatric disorders.

## Materials and Methods

**Strategy**

The protocol of this study was prospectively registered and published in the international prospective register of systematic reviews (PROSPERO) with the number CRD42021268446. Also, the protocol was registered and approved in Persian language at Tehran University of Medical Sciences. AS searched Scopus, ISI Web of Science Core Collection, Embase, MEDLINE, PubMed, CINAHL, Cochrane Library, and Google Scholar (the first 20 citations) databases on June 6, 2021, to obtain the citations required for this study. There was no timespan, language, document type, or publication status limitation in these searches. The search strategy was established using the aim of this review, which was to study SRs (#1) investigating the effectiveness of saffron (#2) for the treatment of neurological and psychiatric disorders (#3), as follows:

#1 - “systematic review” OR “meta-analysis” OR “clinical trials”

#2 - “saffron” OR “crocus”

#3 - depress* OR Alzheimer OR Parkinson OR sexual dys* OR anxi* OR “affective symptoms” OR “psychological symptoms” OR “psychological distress” OR “dementia” OR cogni* OR “Panic Disorder” OR “Post-Traumatic Stress Disorder” OR “Insomnia” OR “Sleep Disorder” OR obsess* OR compuls* OR OCD OR attention-deficit OR ADHD OR hyperactivity OR “Bipolar Disorder” OR phobi* OR Schizophreni* OR “Psychotic Disorder”

The final phrase searched in databases was #1 AND #2 AND #3.

The International Prospective Register of Systematic Reviews (PROSPERO) was manually searched on June 6, 2021, to add related systematic reviews that will be published by the time this review was completed (July 20, 2021). The references of the included SRs were also reviewed manually.

**Inclusion**

Duplicate citations were removed using EndNote X9. AS and AH then screened the citations independently and in parallel and selected the studies, and SA was consulted wherever there was disagreement.

Eligible studies had the following inclusion criteria: (i) SR or meta-analysis (MA) types; (ii) using the words “saffron” or “crocus” in the title, abstract, or keywords; (iii) investigating the effectiveness of *C. sativus* in the treatment of neurological and psychiatric disorders.

The following criteria led to exclusion: (i) *in-vitro* and animal studies; (ii) any type other than SR or MA such as narrative reviews; (iii) lack of explicit reporting of SR or MA methodology; (iv) no clear distinction between non-clinical and clinical studies; (v) inclusion of fewer than two studies with features required in each SR or MA.

There was no restriction on the language of the includable studies. Authors of includable articles were contacted for required data, if any.

**Variables**

Predefined Microsoft Excel 2016 spreadsheets were used for data extraction. AS and AH extracted the data independently and in parallel and sought SA’s opinion in the event of any disagreement. Only SRs or meta-analyses were used to extract the data - not the primary studies that were included.

The following variables were recorded from each included study: first author’s name and publication year, number and design of studies included in, durations of studies, details of the saffron intervention, the patients’ disorder, number of patients participating, how outcomes were measured, the effectiveness of the intervention, analysis results if a MA was performed, adverse events, limitations, and quality of the included SRs or meta-analyses. In addition, SA reviewed the funding for the studies included.

The primary outcome was a report on whether or not saffron was effective in each study. The secondary outcomes were any other related outcomes that SA, the senior author, considered.

AS and AH independently and in parallel used A MeaSurement Tool to Assess Systematic Reviews (AMSTAR) to evaluate the quality of the included studies and used discussion and consultation with SA to resolve discrepancies. AMSTAR is a reliable and valid tool for evaluating the methodological quality of SRs (Shea et al., 2009 ▶). This tool comprises 11 items examining various aspects of the quality. If "yes" is given for each item, it is given a "+1" score, so each study can have a score between zero to 11. Studies with a final score of more than 8 were reported to be high-quality, a final score of 4 to 7 were reported to be medium-quality, and a final score of less than 3 were reported to be poor-quality.

## Results

**Search results**

**Figure 1:**
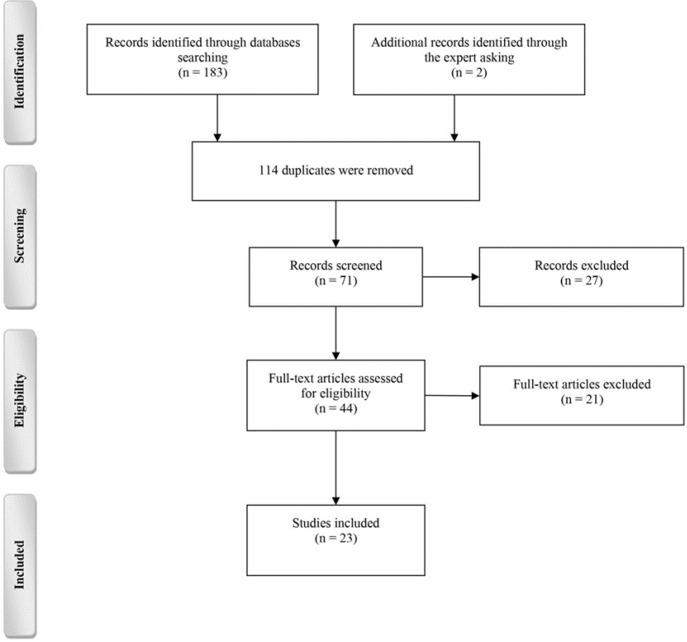
The process of the selection of studies. Reasons for excluding assessed citations are given in Appendix

Figure 1 summarizes the process of reaching the included articles from the primarily collected citations in accordance with the Preferred Reporting Items for Systematic Reviews and Meta-Analyses (PRISMA) guideline. A total of 185 citations were obtained by searching the databases and asking our institution experts, of which, 71 left after the duplicates were removed. After screening, 44 studies remained. At this point, all studies were excluded due to their type. Then, after reviewing the full text of the articles and applying the inclusion and exclusion criteria accurately, 23 studies were included. The characteristics of the included studies are given in Table 1. Reasons to exclude 21 other studies are listed in Appendix.

**Table 1:** Characteristics of the included SRs

**First author,** **Year,** **Country**	**RCT,** **Participants,** **F/U (weeks)**	**ROB,** **Lim**	**Treatment (/D),** **Outcome**	**MA**	**Conclusion**	**Adverse events**
Australia	4, 130-150, 6	NR, SSS/SSD	30 mg saffron, HAM-D	NR	Promising results in the treatment of MDD	NR
	4, 120, 6	NR, SSS/SSD/effects on non-depressed people	30 mg saffron 30 mg petals, Depression symptoms	NR	Promising results in the treatment of MDD	NR
	6, NR, 6-8	mean JS: 4.5, SSS/SSD/lack of extract characterization	30 mg saffron 30 mg petals, HAM-D	NR	Promising results in the treatment of mild to moderate MDD No significant differences between stigma and petals	NS
USA	5, 177 6-8	mean JS: 5, SSS/SSD/single self-report measure/ unknown mechanism of action	30 mg saffron 30 mg petals, HAM-D	Saffron vs. placebo: *M *ES=1.62, 95% CI: 1.10-2.14, p<0.001, *n *= 2; $I^{2}$ =0 Saffron vs antidepressants: *M *ES=-0.15, 95% CI: -0.52-0.22, p=0.42, *n *=3 ES; $I^{2}$=0	Promising results in the treatment of mild to moderate MDD No significant differences between stigma and petals	NS
Australia	6, 230, 6-8	mean JS: 5, SSS/SSD/same research group/other types of MDDs	30 mg saffron 30 mg petals, HAM-D	NR	Promising results in the treatment of mild to moderate MDD No significant differences between stigma and petals	anxiety/ nervousness, increased appetite, nausea, and headache
USA	6-4-1-1, 211-597-50-60, 4-8	mean JS: 5, SSS/SSD/same research group/single self-report measure	30 mg saffron 176.5 mg satiereal saffron 60 mg saffron, HAM-D/DSR/ BW/FSFI/IIEF/SA	NR	Potential positive effects on depression, PMS, SeD, infertility, excessive snacking behavior	NS
Hungary	11 (9), 548, 6-12	? SSS/same country/inhomogeneous patient population	15 mg saffron 30 mg saffron 50 mg saffron 15 mg petals HAM-D/BDI	Saffron vs placebo: *g*=0.891; 95% CI: 0.369-1.412, p=0.001; $I^{2}$ =72.4% Saffron vs antidepressants: *g* = -0.246; 95% CI: -0.495-0.004, p=0.053; $I^{2}$ =0	Reduction in severity of depression (HAM-D and BDI)	NS; caution: allergic reaction and elevated serum parameters
China	7, 316, 6-12	Moderate, SSS/same country/inhomogeneous patient population/same research group	15 mg saffron 30 mg saffron 15 mg petals HAM-D/BDI	Saffron vs placebo: SMD = -1.22; 95% CI: -1.94--0.49, p=0.001; $I^{2}$ =70% Saffron vs antidepressant: SMD=0.16; 95% CI: -2.5-0.57, p=0.44; $I^{2}$ = 42%	Promising results in the treatment of mild to moderate MDD	NS
Iran	21, 927, 4-12	Positive for HAM-D/BDI/PSQI and negative for HAM-A/BAI, Heterogeneity/lack of extract characterization	15 mg saffron 22 mg saffron 28 mg saffron 30 mg saffron 100 mg saffron 30 mg crocin HAM-D/HAM-A/ BDI/BAI/PSQI	BDI (12 effect size): WMD= -4.86; 95% CI: -6.58--3.14, p<0.001; $I^{2}$ =93% BAI (5 effect size): WMD= -5.29; 95% CI: -8.27--2.31; p<0.001; $I^{2}$ =93.9% PSQI (4 effect size): WMD = -2.22; 95% CI: -2.73--1.72; p<0.001; $I^{2}$ =3.6% HAM-D (6) WMD=-1.61; 95% CI: -5.81-2.58, p=0.452; $I^{2}$ = 97.1% HAM-A (3) WMD=-2.74; 95% CI: -5.76-0.27, p=0.074; $I^{2}$ = 90.9%	Reduction in BDI, BAI, and PSQI scores but not HAM-D and HAM-A	NS
China	12, 572, 6-12	Mostly low, SSS/ same country/inhomogeneous patient/	30 mg saffron 30 mg petals, HAM-D/BDI	Saffron vs placebo (mild-moderate) WMD=-5.01; 95% CI: -.43--3.60; p<0.001; $I^{2}$ = 85.3% WMD= -3.35; 95% CI: -3.77--2.93; p<0.001; $I^{2}$ = 98.4% Saffron vs antidepressant (fluoxetine-citalopram) WMD=0.50; 95% CI: -0.29-1.29; p=0.215; $I^{2}$ = 0 WMD=1.14; 95% CI: -1.93-4.21; p=0.466; $I^{2}$=0 Saffron vs placebo (remission-response) RR=1.90; 95% CI: 1.19-3.03; p=0.007; $I^{2}$ =0 RR = 3.41; 95% CI: 1.58-7.34; p=0.002; $I^{2}$ =46.4% Saffron vs antidepressant (remission-response) RR=0.91; 95%CI: 0.66-1.25; p=0.555; $I^{2}$ = 0 RR = 1.03; 95% CI: 0.62-1.71; p=0.897; $I^{2}$ = 0	Promising results in the treatment of mild to moderate MDD	NS
China	1, 40, NR	Low, Not explicitly reported for saffron	30 mg petals, HAM-D	Adverse effect; Saffron vs antidepressant: Risk ratio = 0.68, 95% CI: 0.44-1.06, p= ?	Not explicitly reported for saffron but seemed to be positive?	NS
USA	12, 488, 4-12	NR, Heterogeneity/lack of extract characterization	30 mg saffron 80 mg saffron 100 mg saffron 30 mg crocin 30 mg petals, BDI/BAI/GHQ/MDQ/HAM-D/DSR	NR	Promising results in the treatment of depression and anxiety with minimal risk of serious side effects.	changes in appetite, sexual dysfunction, nausea, headache, insomnia, and tremors
Iran	8, 368, 6-12	NR?Low/moderate, SSS/SSD/heterogeneity/same country/	30 mg saffron	Saffron vs placebo: SMD= -0.86; 95% CI: -1.73-0.00, p<0.01; $I^{2}$ =87% Saffron vs antidepressant: SMD=0.11; 95% CI: -0.20-0.43, p=0.32; $I^{2}$ =15%	Promising results in the treatment of MDD	NS
Iran	6 (3), 384, 1.5-26	NR?Low/moderate Heterogeneity/quality of studies/lack of extract characterization	15 mg saffron 30 mg saffron 50 mg saffron 60 mg saffron 200 mg saffron Topical saffron, ILEF/NPT/SA/ EDITS/HAM-D/ /GEQ/GA/NBT/	Erectile function: MD=5.36; 95% CI: 3.92-6.80, p=0.00; $I^{2}$ =62%; p=0.07 Orgasmic function: MD=1.12; 95% CI: 0.31-1.92, p=0.007; $I^{2}$ =66%; p=0.36 Overall satisfaction: MD=1.23; 95% CI: 0.36-2.10, p=0.005; $I^{2}$ =77%; p=0.01 Satisfaction with intercourse: MD=2.18; 95% CI: 1.22-3.14, p=0.00; $I^{2}$ =63%; p=0.06 Sexual desire: MD = 0.78; 95% CI: -0.01-1.57, p=0.00; $I^{2}$ =0; p=0.37	Promising results in the treatment of men with ED, contradictory results on SA and infertility	NS
Italy	3, 397, 4-12	Mean JS: 4.33 SSS/SSD/lack of extract characterization	30 mg saffron 60 mg saffron Topical saffron, IIEF/SEP/EDITS	NR	contradictory results in the treatment of men with ED	NS
Iran	1, 34, 4	NR, NR	30 mg saffron, FSFI	NR	Effectiveness on the treatment of some fluoxetine-induced sexual problems	NR
Iran	5, 173, 1.5-8	No ROB, SSS/SSD/lack of extract characterization/heterogeneity	30 mg saffron 200 mg saffron Topical saffron, FSFI/ED/desire	SeD: SMD=0.811; 95% CI: 0.356-1.265, p<0.001; SeD subscales SMD=0.493; 95% CI: 0.261-0.724, p<0.001; *Q*=9:981; df = 4; $I^{2}$ = 59.92%; p=0.041	Promising results in the treatment of SeD and its subscales	NR
Iran	2, NR, NR	NR Unknown mechanism	30 mg saffron Topical saffron, ED/tumescence	NR	Promising results in the treatment of men with ED	NR
Australia	23, 1237, 4-12	Mean JS: 4.52, SSS/SSD/same country/Unknown mechanism/lack of extract characterization	30 mg saffron 30 mg crocin 30 mg petals HAM-D/BI/DASS/RCADS/POMS/PANAS	Depression Saffron vs placebo: *g*=0.99; 95% CI: 0.61-1.37, p<0.001 *Q*=71.8; $I^{2}$ = 81.9%; p<0.001 Saffron vs antidepressants: *g* = -0.17; 95% CI: 0.50-0.17, p=0.33 *Q *= 6.16; $I^{2}$ = 35.1%; p<0.19 Saffron as an adjunct: *g* = 1.23; 95% CI: 0.13-2.33, p=0.028 *Q*=27.62; $I^{2}$ = 89.1% Anxiety Saffron vs placebo: *g* = 0.95; 95%CI = 0.27–1.63; p<0.006 *Q* = 44.38; I2 =88.74%; p<0.001	Promising results in the improvement of depression and anxiety symptoms	Headache, nausea, anxiety, constipation, dry mouth, and appetite change
USA	5, 325, 12-48	Low:1/high:1/unclear:3, SSS/high ROB/same country	30 mg saffron, ADAS-cog/MMSE/CDRS-SB/SCIRS/MoCA/WMS-R	NR	Promising results in the treatment of AD	NS
Australia	4, 203, 16-48	Low:1/moderate:2/high:1, SSS/high ROB/diverse tools	30 mg saffron 125 mg saffron, ADAS-cog /CDRS-SB/MMSE/MoCA/NPI/GDS/FAST/FRSSD	NR	Promising results in the treatment of AD and MCI	NS
Iran	8, 306, 4-48	NR	15 mg saffron 30 mg saffron, ADAS-cog /CDRS-SB/MMSE/MoCA/NPI/GDS/FAST/FRSSD?	NR	Promising results in the treatment of AD	NR
USA	2, 124, 6-8	NR, Inclusion of English studies	30 mg saffron, HAM-D/BDI	NR	Promising results in the treatment of PPD	NS

SR: systematic review; RCT: randomized clinical trials; N: number; F/U: follow-up; ROB: risk of bias; Lim: limitation; MA meta-analysis; NR: not reported; SSS: small sample size; SSD: short study duration; HAM-D: Hamilton rating scale for depression; JS: Jadad score; NS: not significant; ES: effect size; PMS: premenstrual symptoms; SeD: sexual dysfunction; DSR: daily symptom report of PMS; BW: body weight; FSFI: female sexual function index; IIEF: international index of erectile function scale; SA: semen analysis; BDI: Beck depression inventory; HAM-A: Hamilton rating scale for anxiety; BAI: Beck Anxiety Inventory; PSQI: Pittsburgh Sleep Quality Index; GCQ: general health questionnaire; MDQ: mood disorder questionnaire; IIEF: international index of erectile dysfunction; NPT: nocturnal penile tumescence test; EDITS: erectile dysfunction inventory of treatment satisfaction; GEQ: global efficacy questionnaire; GA: genetic analysis; NBT: nitro-blue tetrazolium (to examine seminal plasma antioxidant); ED: erectile dysfunction; SEP: sexual encounter profile; FSFI: female sexual function index; DASS: depression anxiety stress scale; RCADS: revised child anxiety and depression scale; POMS: profile of mood states; PANAS: positive and negative affect schedule; ADAS-cog: Alzheimer’s disease assessment scale-cognitive subscale; MMSE: mini mental state examination; CDRS-SB: clinical dementia rating scale-sums of boxes; SCIRS: severe cognitive impairment rating scale; MoCA: Montreal cognitive assessment; WMS-R: Wechsler memory scale-revised; NPI: neuropsychiatry inventory; GDS: geriatric depression scale; FAST: functional assessment staging; FRSSD: functional rating scale for symptoms of dementia; PPD: postpartum depression.

**Quality assessment of the studies**

The quality of the included SRs was assessed using AMSTAR. A significant variation in the AMSTAR score was revealed among these articles. As shown in Table 2, the average AMSTAR score was 6.08 (ranging from 1 to 10). Based on this score, we classified eight SRs as “high-quality SRs,” eleven as “medium-quality SRs,” and four as “low-quality SRs.”

**The effects of saffron on depression**

Thirteen SRs studied the effects of *C. sativus *L. on depression. To the best of our knowledge, in 2007, Sarris performed the first SR that investigated the effects of saffron on major depressive disorder (MDD). He found that this traditional medicine could be used to treat depression (Sarris, 2007 ▶). After that, Morgan et al. also proposed *C. sativus, *both its stigma and its petals, as a promising agent to help people with MDD (Morgan and Jorm, 2008 ▶).

In 2011, Another SR was carried out to evaluate the efficacy of saffron in treating mild to moderate depression (Dwyer et al., 2011 ▶). The authors found that this herb is more effective than placebo in improving Hamilton depression rating scale (HAM-D) scores and has an equal impact on these patients compared to the pharmaceutical antidepressants fluoxetine and imipramine. They also described a randomized controlled trial (RCT) comparing the effectiveness of *C. sativus* stigma and petal, and discovered no significant difference between them.

**Table 2:** AMSTAR scores of the included studies

**First author, year**	**Item 1**	**Item 2**	**Item 3**	**Item 4**	**Item 5**	**Item 6**	**Item 7**	**Item 8**	**Item 9**	**Item 10**	**Item 11**	**Total**	**Quality**
	0	0	1	0	0	0	0	0	0	0	0	1	L
	0	0	1	0	0	0	1	1	1	0	0	4	M
	1	1	1	0	0	1	1	1	0	0	1	7	M
	1	1	1	1	0	1	1	1	1	1	0	9	H
	0	0	1	0	0	1	1	0	0	0	0	3	L
	1	1	1	0	0	1	1	1	0	0	0	6	M
	1	1	1	1	1	1	1	1	1	1	0	10	H
	1	1	1	1	0	1	1	1	1	1	0	9	H
	1	1	1	0	0	1	1	1	1	1	0	8	H
	1	1	1	0	0	1	1	1	1	1	0	8	H
	1	1	1	0	0	1	1	1	1	1	0	8	H
	1	1	1	0	0	1	0	0	0	0	0	4	M
	1	1	1	0	0	1	1	1	1	0	0	7	M
	1	1	1	0	0	1	1	1	1	0	0	7	M
	1	1	1	0	0	1	1	1	1	1	1	9	H
	1	1	1	0	0	1	0	0	0	0	0	4	M
	1	1	1	0	0	1	1	0	1	1	0	7	M
	0	0	1	0	0	0	0	0	0	0	0	1	L
	1	1	1	0	0	1	1	1	1	1	0	8	H
	1	1	1	0	0	1	1	1	0	0	0	6	M
	1	1	1	0	0	1	1	1	1	0	0	7	M
	1	0	1	0	0	1	0	0	0	0	0	3	L
	1	1	1	0	0	1	0	0	0	0	0	4	M
Sum	19	18	23	3	1	20	17	15	13	9	2	140	6.08̽ (M)

AMSTAR: A MeaSurement Tool to Assess Systematic Reviews; L: low; M: medium; and H: high.

Hausenblas et al. in their SR, calculated the Jaded score for the included articles and showed that most of them are high-quality clinical trials (Hausenblas et al., 2013 ▶). They found similar results and revealed that saffron significantly reduced depression symptoms. In an SR implemented in 2014, these positive outcomes of saffron on depression were attributed to its potential serotonergic, neuroendocrine, antioxidant, anti-inflammatory, and therefore, its neuroprotective effects (Lopresti and Drummond, 2014 ▶). Another SR conducted by Hausenblas et al. also confirmed the previous results (Hausenblas et al., 2015 ▶). A high-quality SR and MA on the effectiveness of saffron on depression was implemented by Toth et al. They included 9 RCTs in the final statistical analysis and manifested that *C. sativus* is not less effective than conventional antidepressant drugs and have a significantly better impact on depression symptoms, in comparison with placebo (Tóth et al., 2019 ▶).

Yang et al. also showed that regarding depression symptoms, saffron has an equal impact as synthetic antidepressants (Yang et al., 2018 ▶). They reported a moderate heterogeneity and attributed that to the differences in outcome measures, types of antidepressants as a comparator, and dosage and length of treatment.

The results in a study performed by Ghaderi et al. were controversial in which there was a significant decrease in the Beck depression inventory (BDI) but not in the HAM-D (Ghaderi et al., 2020 ▶). Besides the positive effects of saffron in reducing depressive symptoms, compared with placebo, it has a significant positive effect size in combination therapy with conventional synthetic antidepressants. In the latest SR in this matter, Dai et al. affirmed the previous literature about the efficacy of *C. sativus*. They suggested that saffron could replace pharmaceutical antidepressant agents because they have no significant difference regarding the outcome and adverse effects (Dai et al., 2020 ▶). The results of some other SRs also reaffirmed the foretold statements (Ren et al., 2015 ▶; Yeung et al., 2018 ▶; Khaksarian et al., 2019 ▶).

**The effects of saffron on sexual dysfunction**

It was also found that saffron could improve patients with sexual dysfunction and infertility. One SR has reported that this herb can be effective in treating patients with selective serotonin reuptake inhibitor (SSRIs)-induced erectile dysfunction (Hausenblas et al., 2015 ▶). Saffron also significantly improved the female sexual function index. The authors also mentioned that it was not beneficial compared to sildenafil, in patients with erectile dysfunction.

An SR and MA conducted by Maleki-saghooni et al. revealed that *C. sativus* would increase sperm motility and the number of sperms with normal shape (Maleki-Saghooni et al., 2018 ▶). They also discovered erectile function questionnaire score, satisfaction with intercourse, orgasmic function, sexual desire, and overall satisfaction would become better, using saffron. However, they cautioned that further investigations must confirm this interpretation because of the heterogeneity of these results.

Borrelli et al. described that, unlike sildenafil, saffron has no positive effect on patients with erectile dysfunction (Borrelli et al., 2018 ▶). On the contrary, using oral or topical saffron seemed to be effective in patients with erectile dysfunction. The consumption of saffron seems to positively affect the female sexual function index as a representative of sexual function. It has also been reported that unlike the improvement of arousal, lubrication, and pain scores, saffron does not significantly differ in the desire, orgasm, and satisfaction scores (Molkara et al., 2020 ▶).

Two other SRs were performed on this matter, showed similar results and confirmed that *C. sativus* improves sexual dysfunction (Ranjbar and Ashrafizaveh, 2019 ▶; Solati et al., 2017 ▶).

**The effects of saffron on anxiety**

Yeung et al. narrate that mild to moderate anxiety symptoms can be relieved by using saffron extracts (Yeung et al., 2018 ▶). In the SR performed by Ghaderi et al. the results were debatable; Beck anxiety inventory scores were significantly reduced, unlike the Hamilton Anxiety rating scale scores (Ghaderi et al., 2020 ▶).

Marx et al. demonstrated a sizeable positive effect size for treating with *C. sativus* on decreasing anxiety symptoms. They also noted a significant heterogeneity of the data and publication bias before correction (Marx et al., 2019 ▶).

**The effects of saffron on cognitive disorders**

In an SR implemented by Avgerinos et al. the effects of saffron on cognition were investigated (Avgerinos et al., 2020 ▶). The study showed that patients with Alzheimer’s disease or mild cognitive impairment significantly responded to the treatment with saffron. They reported that using this herb would yield a better score on Alzheimer’s disease assessment scale-cognitive subscale and mini-mental status examination than the patients who received placebo; this impact was similar on patients who received conventional drugs donepezil and memantine. They also warned that due to the lack of RCTs with a low risk of bias, these results should be dealt with cautiously.

Similarly, Ayati et al. noted that *C. sativus* is more effective than placebo and has no significant difference with conventional agents in treating Alzheimer’s disease and mild cognitive impairment. However, no significant difference was seen concerning daily living functions (Ayati et al., 2020 ▶). Talebi et al. performed an SR both on human studies and animal studies (Talebi et al., 2021 ▶). Their results were in accordance with the foretold study. 

**Other effects of saffron**

It has been reported that saffron in women with premenstrual syndrome would reduce depression and anxiety scores (Hausenblas et al., 2015 ▶; Yeung et al., 2018 ▶). Furthermore, it can be helpful for mothers who suffer from postpartum depression as it had a higher positive effect than placebo and relatively equal effect to fluoxetine (McCloskey and Reno, 2019 ▶).

Ghaderi et al. in their SR, also reported that consumption of *C. sativus* could help people to increase their sleep quality. It was revealed using the Pittsburgh sleep quality index (Ghaderi et al., 2020 ▶).

Hausenblas et al. also described that saffron could ameliorate snacking behavior and help people to lose weight (Hausenblas et al., 2015 ▶). Some other positive effects on fatigue, vigor, tension, mood disturbances, confusion, stress, negative affect, social phobia, separation anxiety, and internalization symptoms were also shown (Marx et al., 2019 ▶).

## Discussion

In this study, we reviewed SRs and MAs that investigated the effects of saffron on psychological and cognitive disorders. In summary, treatment with saffron showed promising results and high potential to become an applicable agent in these disorders. Here, we discuss significant limitations of the core studies in the included studies.

Herbal medicines have been used throughout the history of medicine. After the introduction of structured scientific studies and modern medicine, they were repressed for a while, but researchers once again considered them possible agents for prevention and treatment. This reconsideration could be due to their high public approval, satisfying cost-benefit analysis, probable effectiveness, and safety. The new attention to herbal medicine is based on modern scientific processes just like any other approved drug (from purification and understanding the possible mechanism to phase III clinical trials and generalizability) (Eder and Herrling, 2015 ▶).

There are some limitations related to the scientific process for different herbs approval. Controlling the purity standards for saffron is one major drawback. The plant name has been reported in many trials, but the exact substance has not been specified. Newmaster et al. implemented a study, performing DNA barcoding of 44 plant products. They detected significant substitution, contamination, and fillers in these products and reported poor quality in most of them (Newmaster et al., 2013 ▶). In addition to the special importance of the purity of a herb, it is even possible that medicinal plants purchased from the market and used in studies may be mistaken for other plant species (Kahkeshani et al., 2014 ▶).

Another obstacle is that most of the studies do not report the size of the effect and clinical significance and consider the effectiveness as a significant *p*-value. For example, even a small effect can cause statistical significance, while this effect size cannot be detected and distinguished in the clinic (Wasserstein and Lazar, 2016 ▶). Likewise, in many clinical trials investigating the efficacy of saffron, the word “effectiveness” is used after reaching a significant difference with the control group, while this effect will not be detectable in the clinic and means superiority to placebo.

Many clinical trials have shown the safety, tolerability, and potential efficacy of saffron. However, further studies are needed to reevaluate these outcomes. The prescription and consumption of herbal products could have some side effect considerations, such as the interaction of *Hypericum perforatum* (also known as perforate St John’s-wort) with selective serotonin reuptake inhibitors (Davis et al., 2014 ▶).

One great limitation of this study was that most of these SRs were based on the same clinical trials; therefore, they had similar outcomes. It means we should be cautious before interpreting their data. Most of these RCTs have been performed in Iran where saffron has a significant role in traditional medicine, so as mentioned above, further studies must be implemented before any conclusion. It could be a real challenge because saffron’s stigma is very expensive, and providing it could be very hard. 

Saffron has neuropsychotropic effects and is beneficial, safe, and tolerable in treating the mentioned neurological and psychiatric disorders. However, the propensity for herbal medicine should not lead to immature judgments about its prescription and use. Based on the evidence of effectiveness obtained from previous studies, studies on saffron should be continued, but limitations should be considered in conclusions and rectified as much as possible.

## Conflict of interest

The authors have declared that there is no conflict of interest.

## Supplementary material

The online version of this article offers supplementary material.
